# Patterns of Obligation of Care Among Urban and Rural Americans

**DOI:** 10.1093/geroni/igaa057.1152

**Published:** 2020-12-16

**Authors:** Johanna Sosa, Darlingtina Esiaka, Candidus Nwakasi

**Affiliations:** 1 Union College, Schenectady, New York, United States; 2 Union College, Union College, New York, United States; 3 University of Southern Indiana, Evansville, Indiana, United States

## Abstract

In recent years, there is concern about the shortage of adult children who are active participants in the care of their older parents (those 65 years and above). Reviewed studies suggest that attitudes towards the provision of care for older parents differ and may depend on ecological affordances. Particularly, demographic changes due to urbanization shape constructions of obligation to provide eldercare. This study examined patterns of filial responsibility and felt obligation of adult children toward their older parents among rural and urban dwellers in the US. Participants (N= 187) responded to questionnaires assessing filial responsibility, felt obligation to parents, and additional social and demographic characteristics. Results show that rural Americans reported higher levels of filial responsibility and felt obligation to older parents. Also, filial responsibility is associated with the felt obligation for contact with parents, avoidance of conflict with parents, provision of assistance and care, self-sufficiency and independence from parents, and sharing of personal experiences with parents. Our findings indicate the existence of cultural ecological contexts that afford a sense of duty, care, and assistance to older parents. We discuss strategies that acknowledge the relevance of these factors in promoting participation in eldercare.

